# Hand rehabilitation based on the RobHand exoskeleton in stroke patients: A case series study

**DOI:** 10.3389/frobt.2023.1146018

**Published:** 2023-03-22

**Authors:** Patricio Barria, Matías Riquelme, Hannah Reppich, Ana Cisnal, Juan-Carlos Fraile, Javier Pérez-Turiel, David Sierra, Rolando Aguilar, Asterio Andrade, Cristian Nuñez-Espinosa

**Affiliations:** ^1^ Centro de Rehabilitación, Club de Leones Cruz del Sur, Punta Arenas, Chile; ^2^ School of Medicine, University of Magallanes (UMAG), Punta Arenas, Chile; ^3^ Centro Asistencial Docente e Investigación (CADI), University of Magallanes (UMAG), Punta Arenas, Chile; ^4^ Instituto de las Tecnologías Avanzadas de la Producción (ITAP), University of Valladolid, Valladolid, Spain

**Keywords:** hand exoskeleton, manual function assessment, rehabilitation technology, stroke, user satisfaction

## Abstract

**Introduction:** The RobHand (Robot for Hand Rehabilitation) is a robotic neuromotor rehabilitation exoskeleton that assists in performing flexion and extension movements of the fingers. The present case study assesses changes in manual function and hand muscle strength of four selected stroke patients after completion of an established training program. In addition, safety and user satisfaction are also evaluated.

**Methods:** The training program consisted of 16 sessions; two 60-minute training sessions per week for eight consecutive weeks. During each session, patients moved through six consecutive rehabilitation stages using the RobHand. Manual function assessments were applied before and after the training program and safety tests were carried out after each session. A user evaluation questionnaire was filled out after each patient completed the program.

**Results:** The safety test showed the absence of significant adverse events, such as skin lesions or fatigue. An average score of 4 out of 5 was obtained on the Quebec User Evaluation of Satisfaction with Assistive Technology 2.0 Scale. Users were very satisfied with the weight, comfort, and quality of professional services. A Kruskal-Wallis test revealed that there were not statistically significant changes in the manual function tests between the beginning and the end of the training program.

**Discussion:** It can be concluded that the RobHand is a safe rehabilitation technology and users were satisfied with the system. No statistically significant differences in manual function were found. This could be due to the high influence of the stroke stage on motor recovery since the study was performed with chronic patients. Hence, future studies should evaluate the rehabilitation effectiveness of the repetitive use of the RobHand exoskeleton on subacute patients.

**Clinical Trial Registration:** https://clinicaltrials.gov/ct2/show/NCT05598892?id=NCT05598892&draw=2&rank=1, identifier NCT05598892.

## 1 Introduction

Multiple situations can compromise the motor function of people, such as accidents or illnesses. Within the latter, cerebrovascular accidents (CVA) are one of the main causes of mortality, morbidity, and disability globally (Collaborators, 2019). Numerous risk factors for suffering a stroke are prevalent in our population, such as hypertension and diabetes (Leyton Pavez et al., 2019; Sepúlveda-Contreras, 2021).

About 75% of people who have a stroke, experience paresis of the upper extremities, especially their hand (Fischer et al., 2007; Rathore et al., 2022). Among the main physical consequences is the reduction of strength and spasticity (Thibaut et al., 2013). The initial rehabilitation should be performed in a dedicated stroke unit and must be intensive. It is recommended that early rehabilitation consists of 45-min sessions for a minimum of 5 days per week (Dworzynski et al., 2015). Early intervention is also of great importance in order to maximize functional motor recovery and independence after a stroke. It has been proven that early and intensive rehabilitation is associated with a better functional outcome (Klijn and Hankey, 2003; Phipps and Cronin, 2020). The potential motor recovery is generally achieved during the 6 months after the stroke. Recovery is complex and it may occur through a combination of spontaneous neurological recovery and rehabilitation process (Langhorne et al., 2011). There is a period of 1–3 months after suffering a stroke where both spontaneous and intervention-mediated motor recovery is maximal due to the enhanced brain plasticity (Zeiler, 2019).

Motor recovery depends on the severity of the stroke and therefore, the initial grade of paresis. For instance, 6 months after an acute stroke, only 38% of patients were found to have some dexterity in the paretic limb and 12% of patients achieved full functional motor recovery (Hendricks et al., 2002). Optimal prediction of functional recovery can be made within the first 4 weeks of the stroke episode (Kwakkel et al., 2003). Around 90% of stroke patients recover motor functions in a proportional manner with respect to the initial level of impairment, achieving approximately 70% of their maximal potential recovery within 3–6 months from suffering the stroke (Prabhakaran et al., 2008).

Based on the above, it is essential to understand the mechanisms that promote the best possible recovery of patient’s motor activity and sensory feedback (Rossini et al., 2003). Different studies have shown that repetitive, goal-directed functional activities have been associated with positive changes in brain activity (Nudo et al., 1996; Jones, 2000) and thus in recovery. Indeed, repetitive task rehabilitation improves hand motor performance of stroke patients, especially increasing the range of motion (ROM) and strength (Bütefisch et al., 1995; Levy et al., 2001; Sterr and Freivogel, 2003).

However, traditional rehabilitation is costly because it requires a lot of time of the rehabilitation specialist (Frick and Alberts, 2007). A rehabilitation robotic system that allows the patients to perform repetitive rehabilitation exercises without the continuous assistance of the therapist, would make physical therapy more accessible and affordable, increasing the potential for better outcomes (Panagiotis et al., 2015).

Due to the aging of society, there is a need for innovation in rehabilitation technologies that allow one therapist to assist multiple patients at the same time. In the coming decades, the use of these robotic rehabilitation devices will be necessary if chronic diseases of the musculoskeletal and nervous systems are to be adequately treated, as there will be more patients and fewer therapists (Ates et al., 2017).

Rehabilitation therapies have made great strides in recent years, where information technology, robotics, and the design of exoskeletons have been key elements in new therapies (Pelier et al., 2021). These allow the practitioner to have more information to evaluate the response to certain exercises and then adapt the therapy mechanism or exoskeleton to the biomechanics, requirements, and demands of the patient (Gil et al., 2022). Additionally, it allows the performance of more prolonged and repetitive exercises compared to those offered in traditional rehabilitation therapy (Mancisidor et al., 2018).

Rehabilitation therapies based on virtual reality have been found less boring than conventional therapies (Cisnal et al., 2022). They provide motivation to post-stroke patients (Loureiro et al., 2004; Flores et al., 2009), resulting in a more effective rehabilitation because patients are more likely to continue with therapy (Cameirao et al., 2007; Corbetta et al., 2015). The ideal game in stroke rehabilitation should be based on activities of daily living (Sveistrup, 2004; Kwakkel et al., 2008; Bo Nielsen et al., 2015). Furthermore, providing adequate feedback has been shown to strengthen the patient’s attitude in a positive way. In general, the use of virtual reality in rehabilitation has been found beneficial for stroke patients (Crosbie et al., 2008; Subramanian et al., 2013).

In this line of work, there are multiple assistive robotic platforms such as hand exoskeletons, gloves, or end-effector devices. They usually have multiple sensors in their design that report more precisely on the execution of the movement, compared to other more traditional intervention processes. With these new technologies, the range and type of movement to be performed can be programmed with greater precision, obtaining better results in therapy. Furthermore, they integrate different types of feedback, such as those based on biosignals, to enhance user performance (Cisnal et al., 2023).

Clinical studies have shown significant improvement in hand motor when performing intense repetitive movements assisted by robotic devices (Kutner et al., 2010; Carmeli et al., 2011; Ueki et al., 2012), concluding that there is an advantage of robotic devices because they facilitate independent rehabilitation with the possibility of increased training repetitions and patient motivation (Kwakkel et al., 2008; Rietman et al., 2014).

A study carried out by (Gomez Rendon et al., 2016), showed that robotic hand orthosis can be considered as a potential application for rehabilitation and assistance of those patients with hand disabilities. Additionally, this technology efficiently complements conventional rehabilitation therapy in people who present partial or total functional loss of the hand. In another study conducted by (Sale et al., 2012), the Amadeo robot (TyroMotion, Austria) was used for hand rehabilitation with hospitalized stroke patients. The rehabilitation consisted of 20 treatment sessions during 4 consecutive weeks (5 days per week), with a duration of 40 min per session. The results of the statistical analysis showed a positive effect of the robot-assisted approach in the early phase and suggested that spasticity management is more effective if the rehabilitation treatment begins during the acute phase.

Vanoglio et al. (2016) conducted a clinical trial on the Gloreha–Hand Rehabilitation Glove (Gloreha IDROGENET, Italy) to validate its effectiveness. The study includes a treatment group and a control group, including patients who suffered a stroke 17.8 ± 7.9 and 15.2 ± 6.8 days after the intervention. The rehabilitation consisted of 30 sessions, each one 40 min. They conducted the Nine-Hole Peg Test (9-HTP) to assess the grip and pinch force. The results showed that both groups recovered some motor functions. However, only the group treated with Gloreha showed significant improvements. On the contrary, no significant differences in functional motor recovery were found between the robot-assisted and the classical occupational therapy when comparing subacute stroke patients (all patients suffered the stroke less than 4 months before the study) (Orihuela-Espina et al., 2016).

Additionally, Koumpouros, (2016) conducted a systematic review to identify existing frameworks for the subjective assessment of rehabilitation and assistive devices. This review detected a great gap in the subjective assessment of the assistive rehabilitation devices since only 6.1% of in selected studies used validated measures. Evaluating user satisfaction is essential to design robotic devices that meet the needs of the intended end users.

Rehabilitation using robotic devices should be safe and user-approved. Furthermore, they should also guarantee an intensive and task orientated process, at a relatively moderated cost, where it is possible to assist with precise forces, which are potentially effective in strengthening muscles, improving ROM and motor coordination (Aggogeri et al., 2019). As these technologies are still incipient, it is necessary to increase the existing evidence on them, in order to carry out evidence-based therapies, and with it also design protocols for implementation at scale.

The RobHand is a new hand exoskeleton for neuromotor rehabilitation that assists patients in performing flexion and extension movements of the fingers (Moreno-SanJuan et al., 2021). The aim of this clinical trial is to determine the changes in manual function and hand muscle strength in stroke patients after completion of an established training program. As not only motor recovery is important when evaluating rehabilitation robots, safety, and user satisfaction tests were also conducted.

## 2 Methods

### 2.1 Preliminar pilot study

Prior to conducting this clinical trial, a pilot study was performed by the Neurotechnologies Research Group at the Movement Analysis Laboratory of Club de Leones Cruz del Sur Rehabilitation Corporation. This pilot study was conducted with four healthy subjects who were evaluated on their manual hand function before and after participating in 16 rehabilitation session, using RobHand.

The pilot study showed that user satisfaction of RobHand was favorable, observing the absence of pain after using the equipment. In addition, no lesions were reported or observed after the removal of the exoskeleton.

Due to the absence of adverse events during the training sessions, where each participants completed the full duration of each session, the pilot study allowed the publication of research methods and protocols, therefore enabling the start of the clinical study.

A report is provided as supplementary material. It is significant to emphasize that this report was based on a pilot study, therefore, no changes in manual function could be observed as all participants had normal manual function.

### 2.2 Setting

The present clinical trial was set at the Rehabilitation Center Club de Leones Cruz del Sur in Punta Arenas (Chile).

### 2.3 Patients

The study participants were chronic stroke patients specifically selected by the researchers as they represent the majority of patients at Corporación de Rehabilitación Club de Leones Cruz del Sur. No control group was used in this study.

**FIGURE 1:**
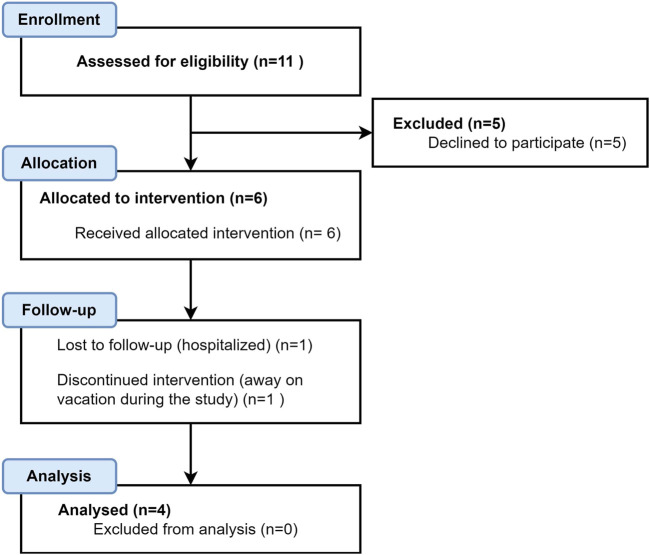
CONSORT (Consolidated Standards of Reporting Trials) flow diagram.

Of the eleven selected patients that were eligible for the study, five declined and one additional patient was excluded due to non-completion of the rehabilitation program. One additional patient who completed the program, did not complete the follow-up interview and therefore the results were also not included. Hence, four participants completed the study and only their results were analyzed (Figure 1).

The four participants, 3 men and 1 woman, had an average age of 60.8 years (±4.9 years). For privacy of patient data, each was assigned a six-digit alphanumeric code, whose participant assignment was only know to the principal investigator. Demographic data of the participants is shown in Table 1.

**TABLE 1:** Stroke participant demographics.

ID	Gender	Age	Affected	Months	Social
Participant			laterality	since stroke	status
G9GJCG	F	56	Left	17	Married
HAJNFU	M	62	Left	181	Married
EY7E6F	M	67	Left	73	Married
2WQT89	M	58	Left	22	Married

#### 2.3.1 Eligibility criteria

To be included in the study, it was necessary to fulfill the following criteria: patients included had to be at least 18 years old, be an active patient of the Club de Leones Cruz del Sur Rehabilitation Corporation, suffered at least one stroke, possess an adequate level of consciousness to follow orders, agree to voluntarily participate in the study, and have signed the informed consent form.

#### 2.3.2 Exclusion criteria

The exclusion criteria for the Stroke Group were history of comorbidity in the central nervous system, pain in the upper extremity (hand, forearm, arm, and/or shoulder) and patients who did not sign the informed consent form.

### 2.4 Confidentiality

Only institution employees, co-researchers, and ethics committee members get access to the participants records. To ensure the privacy of patient data, each one was assigned a six-digit alphanumeric code, so the participants identities were concealed in any research related publications.

### 2.5 Rehabilitation system

**FIGURE 2:**
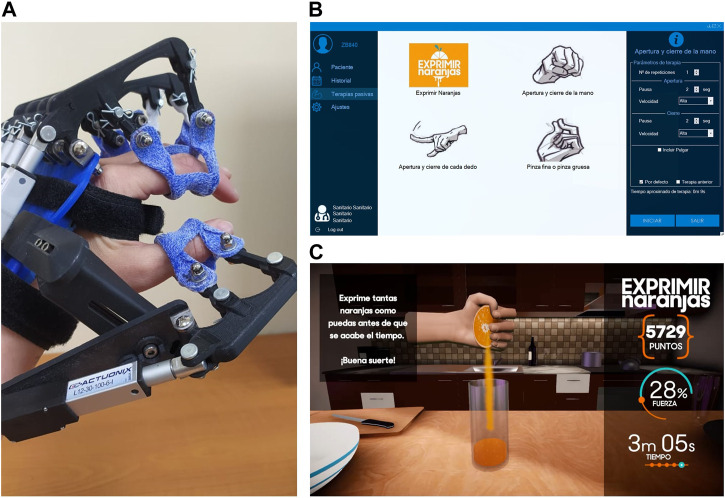
RobHand rehabilitation platform. **(A)** RobHand exoskeleton **(B)** Tab for selection and configuration therapies **(C)** Squeeze oranges therapy.

The RobHand (Robot for Hand Rehabilitation) is an exoskeleton-type electromechanical device, which is attached to the patient’s hand and provides assistance for performing different types of finger movement rehabilitation therapies (Figure 2A). The RobHand platform was developed by the University of Valladolid, Spain (Cisnal et al., 2018).

The exoskeleton is composed of five independent subassemblies that are placed on a platform which is located on the back of the hand, with the exception of the thumb subassembly which is mounted on a separated module connected to the hand support platform through a linkage device. Each subassembly includes a linkage underactuated mechanism that transmits the movement of the linear actuator L12-30-100-6-I (Actuonix Motion Devices Inc., Victoria, BC, Canada) to a double-ring. The exoskeleton is attached to the proximal and distal phalanges through the double-rings. Hence, the proposed linkage mechanism allows to control flexion and extension angles of the metacarpophalangeal (MCP) and proximal interphalangeal (PIP) joints of each finger with a single linear actuator. Therefore, the motion of the MCP and PIP joints of each finger is kinematically coupled. The use of this linkage mechanism reduces size, weight and cost of the exoskeleton due to the reduction in the number of actuators used. Furthermore, the mechanical structure has been optimized to cover the ROM of a healthy human hand with a 30 mm stroke actuator. More precisely, the index finger can reach a maximum hyperextension movement of 8° and −5° at the MCP and PIP angles and a maximum flexion of −63° and −76° at the MCP and PIP joints, respectively (Moreno-SanJuan et al., 2021).

The double-rings ease the donning and doffing procedure of the exoskeleton, which is critical for patients suffering from hand spasticity. The custom double-rings made of ORFITCAST thermoplastic material are placed on each finger, and then they are jointed to the corresponding linkage mechanism. Furthermore, due to the special mobility characteristics of the thumb, the exoskeleton integrates a simple mechanism that provides an easy adaptation of the thumb subassembly to the exoskeleton and therefore, allowing to achieve comfortable movements of the thumb, regardless its size and length. The hand exoskeleton is adjusted on the hand of the patient using two Velcro straps joint to the hand support platform and placed around the hand palm and wrist. A flexible splint is used to secure the position of the patient’s wrist. The hand exoskeleton structure was manufactured using 3D printing of PLA and weighting approximately 450 gr.

The five linear actuators are independently controlled by a TMS300F28069M microcontroller (Texas Instruments, Texas, EEUU) using a 0–5 V interface and are powered by 6 V DC. A custom-made motor driver PCB allows to properly control the actuators with the microcontroller and provides a 20-pin single connector to interface the hand exoskeleton to the electronic box. All the electronics are housed in a 3D-printed electronic box. The electronic box has a reset button, a 6 V DC jack power connector, an on/off switch, a visual indicator light, and a kill-switch for security reasons (Cisnal et al., 2021).

The RobHand platform allows patients to perform passive training exercises, which involve the continuous repetition of finger flexion and extension movements at three predefined velocities. The hand exoskeleton is controlled by a windows-based application. Before starting a rehabilitation session, the therapist must log in to the application using their credentials and select the patient among those registered in the application or create a new one if it does not exist. Then, the therapist must select which type of exercise to perform and configure it by specifying the number of repetitions and velocity (low, medium, and high) (Figure 2B). Four types of rehabilitation exercises are available: i) Squeeze oranges (Figure 2C)—flexion and extension of the hand fingers with the aim making orange juice ii) Hand opening and closing—flexion and extension of the five hand fingers simultaneously iii) Fingers opening and closing—flexion and extension of hand fingers individually iv) Pinch grip and precision grip—flexion and extension of the thumb against the four fingers or against the index finger. The therapist can also define a comfortable ROM (maximum and minimum MCP angles for each finger) for performing therapies according to the patient’s residual skill (Cisnal et al., 2022). All data is store in a local database and the therapist can review the therapy history of each patient.

### 2.6 Intervention

**FIGURE 3:**
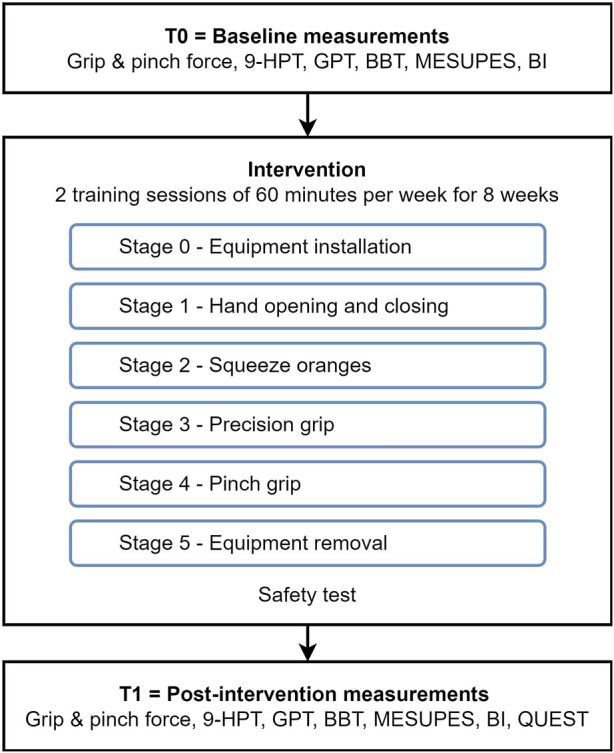
Flow diagram of the stages of the intervention and baseline and post intervention manual function tests.

The rehabilitation program consisted of two training sessions of 60 min per week for a total of 16 sessions using the RobHand exoskeleton on the impaired hand. Subjects were instructed not to resist the assistance motion of the exoskeleton. These sessions occurred between July 2021 and October 2021. Each training session was divided into six consecutive stages (Figure 3).

#### 2.6.1 Stage 0—Equipment installation:

The participant is asked to sit in an ergonomic chair with their arm flexed at 90°, and forearm resting on a semisoft wedge on a table, to leave the hand free for the movements performed by the exoskeleton. The installation, configuration, and positioning of the exoskeleton was performed by an occupational therapist with experience in robotic rehabilitation. This stage took approximately 5 min.

#### 2.6.2 Stage 1—Hand opening and closing:

**FIGURE 4:**
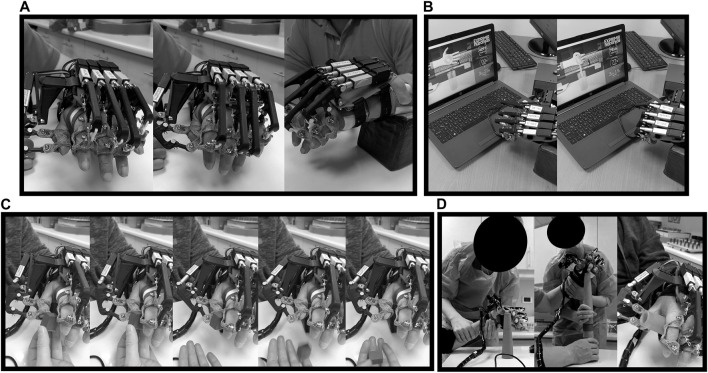
Participant performing a training session: **(A)** Hand opening and closing (stage 1), **(B)** squeeze oranges (stage 2), **(C)** precision grip (stage 3) and **(D)** pinch grip (stage 4).

The training began when the researcher gave the start indication, at which point the RobHand performed the opening-closing actions for the participant (Figure 4A). 75 repetitions per session were completed with a two second break in between. The stage took five minutes and seven seconds to complete.

#### 2.6.3 Stage 2—Squeeze oranges:

This exercise is performed in sync with audiovisual material, showing how a hand is squeezing oranges while the RobHand is performing the movement with the hand of the patient (Figure 4B). 19 repetitions per session were completed with four seconds break in between. The stage took four minutes and eleven seconds to complete.

#### 2.6.4 Stage 3—Precision grip:

During this stage a preconfigured movement, provided by the RobHand software, was used. The RobHand guided the patients thumb and index finger to grab a small cube, simulating fine pincer movements. The instructions given by the evaluator were: “I am going to ask you to grab this cube (the instructor held the cube near the hand of the patient) using your thumb and index finger and place it in my open hand.” (Figure 4C). 75 high speed repetitions were completed with two seconds break in between each closing and opening movement. The stage took five minutes and eleven seconds to complete.

#### 2.6.5 Stage 4—Pinch grip:

In this stage, the semisoft wedge was removed and instead the evaluator moved the patient’s arm from one point of the table, across their body to another point, while stacking cones (Figure 4D). The evaluator gave the following instruction: “I am going to ask you to take each one of the cones and stack them with my help (supporting the arm), on the other side of the table”. 20 repetitions were completed with four seconds in between each pincer movement. The stage took five minutes and eleven seconds to complete.

#### 2.6.6 Stage 5—Equipment removal:

The removal of the exoskeleton was performed by an occupational therapist with experience in robotic rehabilitation. After the removal of the equipment, the patient was checked for adverse events like pressure points, skin problems or pain. This stage took five minutes to complete.

### 2.7 Assessments

The evaluation of the device safety, the manual function and user satisfaction was carried out by professionals from the movement analysis laboratory of the Club de Leones Cruz del Sur rehabilitation corporation (Punta Arenas, Chile). Different instruments were used for manual function assessment: dynamometry for grip and pinch strength assessment, Nine Hole Peg Test (9-HPT), Grooved Pegboard Test (GPT), Box and Blocks Test (BBT), Motor Evaluation Scale for Upper Extremity in Stroke Patients (MESUPES) and Barthel Index (BI). Manual function assessment was performed before (participant’s baseline or T0) and after the intervention with the RobHand robotic exoskeleton (post-intervention or T1). Safety tests were carried out after each training session. Furthermore, all patients fulfilled the Quebec User Evaluation of Satisfaction with Assistive Technology (QUEST 2.0) questionnaire after the intervention (T1). The timestamps at which each test was performed are shown in Figure 3.

#### 2.7.1 Safety evaluation

The clinician asked the patient after every training session whether they had suffered pain or localized fatigue. The clinician also visually checked whether there were cutaneous lesions or pressure zones present on the skin.

#### 2.7.2 Grip strength assessment

**FIGURE 5:**
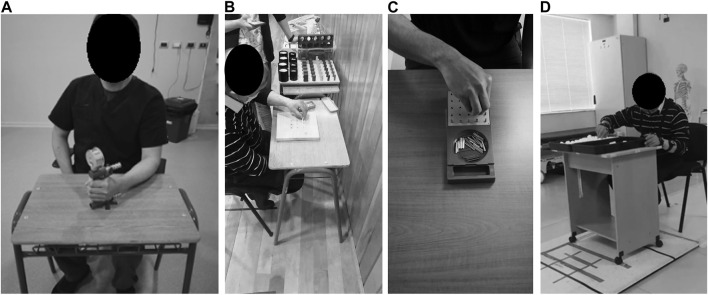
Participants performing manual function tests: **(A)** Grip dynamometry, **(B)** 9-HPT, **(C)** GPT and **(D)** BBT.

A Jamar hydraulic hand dynamometer (JAMAR, hydraulic, model 12-0600, 5 lbs or 2 kg gradations, Pennsylvania, United States) was used to measure grip strength, which allows evaluation of forces up to 200 lbs (90 kg). This test evaluates the functional integrity of the upper extremity through the force exerted when squeezing the hand and therefore, to identify the loss of physiological muscle function (Armando et al., 2012). The patient is asked to grasp the resistance of the handle, place his shoulder in abduction and with neutral rotation (Figure 5A). Additionally, the elbow must be flexed at 90° and with the forearm in a neutral position, the wrist between 0° and 30° dorsiflexion, and between 0° and 15° ulnar deviation (Ong et al., 2017). Grip measurements were repeated three times and the average value is reported.

#### 2.7.3 Pinch strength assessment

The force exerted with the index finger and thumb is assessed using a Jamar hydraulic pinch gauge (JAMAR, hydraulic pinch, model 12-0601, gradations 1 lbs o 0.5 kg gradations, Pennsylvania, United States), which allows evaluation of forces up to 50 lbs (30 kg). The measurement is standardized in its procedure according to publications of literature (Gilbertson and Barber-Lomax, 1994). The thumb was positioned on top of the pinch gauge’s force pad and the index fingertip was positioned underneath. The researcher supported the gauge and asked the participant to grip and pinch with their maximum strength. The measurement was performed three times and the average results are reported.

#### 2.7.4 Nine hole peg test (9-HPT)

9-HPT seeks to evaluate the dexterity of the fingers (Oxford Grice et al., 2003), where a board and nine pegs are used. The test is timed, and the patient must place the nine pegs in marked holes on the board and then remove all of them, using the impaired hand (Figure 5B).

#### 2.7.5 Grooved pegboard test (GPT)

GPT allows to evaluate a variety of psychomotor skills, including fine motor skills, motor speed and hand-eye coordination. During the test 25 pegs, with a key on one side, must be placed on a board with 25 holes, each placement requiring the pegs to be rotated differently to match the holes orientation (Tolle et al., 2020). The test is performed with the affected upper limb and is timed. If the tested person is not able to match all pegs in 5 min, the tester counts the number of matched pegs on the board (Figure 5C).

#### 2.7.6 Box and blocks test (BBT)

BBT is used to evaluate the unilateral gross manual dexterity. It consists of a wooden box divided into two compartments by a partition, as well as 150 blocks. The participant is asked to move the largest number of blocks, one at a time, from one compartment to the other, in a time of 1 min. Before carrying out, standardized instructions should be given to the patients, instructing them that their fingertips should cross the partition when transferring the blocks, and that they do not need to pick up the blocks that could fall out of the box (Figueiredo, 2011). Furthermore, the box on a table must be in front of the patient, oriented longitudinally and in line with the participant, with the compartment that contains the blocks, parallel to the hand to be evaluated (Figure 5D). Before the evaluation, a trial period of 15 s is allowed for each hand to be evaluated. A higher score indicates a better manual dexterity.

#### 2.7.7 The motor evaluation scale for upper extremity in stroke patients (MESUPES)

This scale measures the quality of hemiparetic arm and hand movement performance in patients with stroke disabilities (de Winckel et al., 2006). It consists of 17 items divided into two subscales: arm (eight items, with scores from 0 to 5) and hand (nine items, with scores from 0-2). The total score for this test ranges from 0 to 58 points, where higher scores correlate with greater autonomy. The evaluation of the hand function is divided into two parts, in the first part (six items) patients are instructed to perform specific movements of the hand and fingers, being graded by the ROM. In the second category (three items) they are asked to perform functional tasks and are scored by the correct orientation of the hand and fingers during execution.

When evaluating the arm, the movements are scored in three consecutive phases and for the evaluation of the first four items these must be performed in the supine position; all other elements are performed in a seated position with hips and knees flexed to 90° and elbows on the table. The patient cannot be evaluated if they cannot maintain an upright position for tasks in a sitting position. The therapist must wait until the tone normalizes before beginning a new task. If the patient is unable to achieve a relaxed starting position, a score of 0 is given for the item.

#### 2.7.8 The barthel index (BI)

BI is an ordinal scale, widely used in the population with stroke. It allows to measure the performance of daily life by rating ten daily life activities such as feeding, mobility or self-care (Gomez Rendon et al., 1965). Each variable has different values, which are based on the physical assistance required to perform each task. BI is calculated by adding up all individual scores. The total maximum score is 100, which determines the degree of dependency (Table 2).

**TABLE 2:** Degrees of dependency according to the obtained results applying the Barthel Index.

Result	Degree of dependency
≤20	Total
20-35	Serious
40-55	Moderate
≥60	Mild
100	Independent

#### 2.7.9 Evaluation of user satisfaction with assistive technologies in quebec (QUEST 2.0)

It is a self-administered questionnaire that allows to consider personal aspects related to the use of a device, in order to assess user satisfaction when using an assistive device. This can be applied to adolescents, adults, and seniors (Demers et al., 2000). This questionnaire consists of two sub-scales; the first evaluates eight aspects related to the assistance team, while the second evaluates four aspects related to the services provided while the participant uses the device. The responses are rated using the Likert Scale (from 1 to 5), where 1 means not at all satisfied and 5 means very satisfied.

### 2.8 Statistical analysis

Statistical analyses were performed using the R Statistical Software with the alpha level set to 0.05 for statistical significance. The results coming from the performed tests were analyzed using the non-parametric statistical test of Kruskal–Wallis, which is equivalent of the one-way analysis of variance (ANOVA). We select a non-parametric test due to the low number of samples (*n* = 4). Kruskal–Wallis tests were performed to evaluate changes in baseline (T0) and post-intervention (T1) results of the performed manual function test (df = 1). Chi square statistics along with unadjusted *p*-values are reported for each test.

## 3 Results

The results of this intervention have been divided into three segments: safety tests, manual function tests and satisfaction analysis.

### 3.1 Safety evaluation

The results of the application of the safety monitoring scale for users of support technologies after the use of the RobHand exoskeleton in subjects with stroke (Table 3) evidenced the absence of significant adverse events during the study period, such as skin lesions or fatigue. Nevertheless, all the participants presented pressure points on the skin that were present after the removal of the exoskeleton, and which disappeared a few minutes after its removal. Regarding to pain events, there were reported four pain events, but in a distal area to the use of the device, in the shoulder joint.

**TABLE 3:** Results of the safety test after using the RobHand: Presence of pain, cutaneous lesions, skin pressure zones and localized fatigue.

ID	Pain	Cutaneous	Skin	Localized
Particpant		lesions	pressure zones	fatigue
G9GJCG	0/16	0/16	16/16	0/16
HAJNFU	2/16	0/16	16/16	0/16
EY7E6F	1/16	0/16	16/16	0/16
2WQT89	1/16	0/16	16/16	0/16

### 3.2 Manual function tests

**FIGURE 6:**
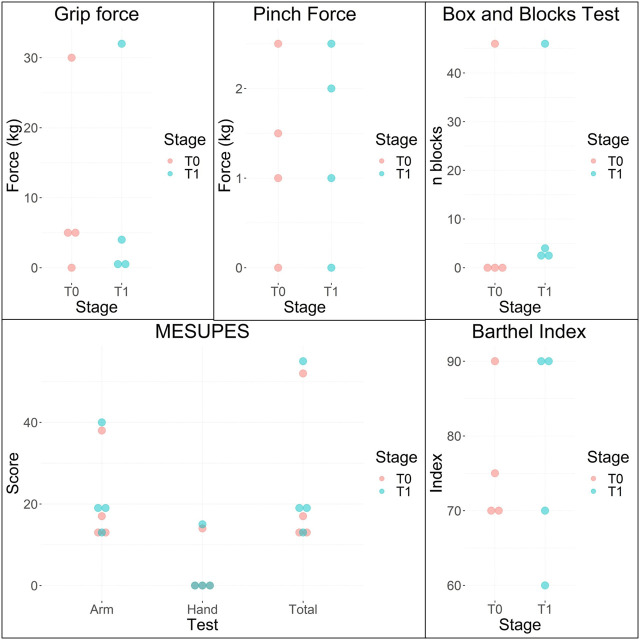
Baseline (T0) and post intervention (T1) results of grip dynamometry, pinch dynamometry, BBT, MESUPES and BI.

The manual function tests were applied to all participants, except for the 9-HPT and GPT. Both require fine motor skills and could not be implemented in all the study patients due to their degree of affection of the fine motor function. Only one participant (ID Patient = 2WQT89) underwent these evaluations. Results coming from the five performed manual tests at the pre-intervention stage (T0) and post-intervention stage (T1) are shown in Table 4 and Figure 6. Kruskal–Wallis Test was conducted to examine the differences on the results according to the intervention stage: baseline results (T0) and the results obtained after 16 training sessions (T1).

**TABLE 4:** Baseline results (T0) and post-intervention results (T1) of the performed manual function tests: grip, pinch, BBT, MESUPES and BI.

ID patient							MESUPES							
	Grip		Pinch		BBT		Total		Arm		Hand		BI	
	(kg)		(kg)		(n blocks)		(score)		(score)		(score)		(index)	
	T0	T1	T0	T1	T0	T1	T0	T1	T0	T1	T0	T1	T0	T1
G9GJCG	0.0	1.0	1.0	1.0	0	2	13	13	13	13	0	0	70	60
HAJNFU	5.0	4.0	2.5	2.5	0	4	17	19	17	19	0	0	90	90
EY7E6F	30.0	32.0	0.0	0.0	46	46	52	55	38	40	14	15	75	90
2WQT89	5.0	0.0	1.5	2.0	0	3	13	19	13	19	0	0	70	70

No significant differences (Chi square = 0.1920, *p* = 0.6612) were found among T0 and T1 for the measured grip force. Significant differences were also not found in the measured pinch force (Chi square = 0.0216, *p* = 0.8831). Regarding to the BBT, only one participant (ID Patient = EY7E6F) was able to grasp and transfer blocks in T0, while the four participants were able in T1. No statistically significant differences were observed in the number of blocks transferred from one compartment to the other in one minute when comparing T0 with T1 (Chi square = 1.7943, *p* = 0.1804). No significant differences were found in the MESUPES test when comparing T0 with T1 for total (Chi square = 0.7974, *p* = 0.3719), arm (Chi square = 0.7974, *p* = 0.3719) and hand (Chi square = 0.0357, *p* = 0.8501) scores. Regarding to the reported Barthel indexes, no significant differences were observed (Chi square = 0, *p* = 1.0000).

The only patient with fine motor skills performed the 9-HPT in 55.22 s at T0 and 58.87 s at T1. Regarding to GPT, the patient was able to enter 9 pegs in five minutes during T0, while in T1 he entered 10 in the same time. The subject worsened the 9-HPT results with an increase of 3.65 s. In contrast, the performance in the GPT improved and one more peg was interceded in the same time.

### 3.3 Satisfaction analysis

**FIGURE 7:**
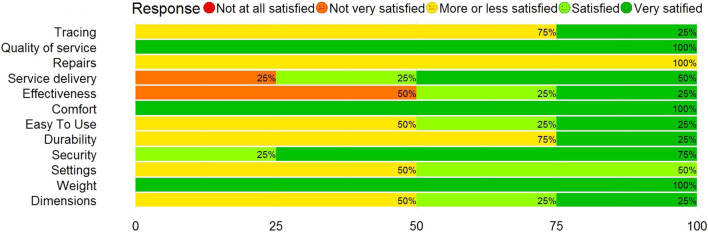
Results of the QUEST questionnaire.

After completing 16 training sessions, the QUEST questionnaire was performed by all study participants (Figure 7). The participants were ‘satisfied’ with the RobhHand exoskeleton as rehabilitation device, giving an average score of 4.00 ± 0.73. Weight, comfort, and quality of services were given the highest rating (5.00), while the repairs item was given the lowest ranking (3.25) followed by tracing (3.50).

In relation to the equipment dimensions, two considered themselves more or less satisfied with them, one quite satisfied and one very satisfied. The four users were very satisfied with the weight. Regarding the adjustments, that is, if the team adapts to its context, two participants were neutral and two quite satisfied. In the case of the security variable, three users felt very satisfied with the use of the hand exoskeleton, while the other one was quite satisfied. In relation to durability, three were neutral and one strongly satisfied. In relation to ease of use, that is, if the subjects consider it easy to use the equipment, three were satisfied and one strongly satisfied. Regarding the comfort of the equipment, the four participants were strongly satisfied. Regarding the effectiveness of the team, one user was quite satisfied, one was strongly satisfied and the other two were not very satisfied. This means that half of the group considered a high effectiveness in the use of the equipment in relation to the objective set in the exercise. The levels of satisfaction regarding the delivery of service during the use of the equipment showed that two were highly satisfied users, one was quite satisfied, and the other was not very satisfied. Satisfaction levels with respect to the repairs performed on the equipment during the therapy period resulted in all four users being highly satisfied. Regarding the variable quality of professional services, the four declared themselves strongly satisfied. This means that there is a high degree of appreciation for the service provided by professionals during the use of the equipment. Finally, regarding the monitoring of the team of professionals in relation to the use of the equipment, three were neutral and one strongly satisfied.

## 4 Discussion

The objective of this research was to determine the effects of rehabilitation with the RobHand exoskeleton on manual function, safety aspects and satisfaction of four chronic stroke patients at the Corporación Club de Leones Cruz del Sur (Punta Arenas, Chile).

### 4.1 Safety evaluation

The performed safety tests has demonstrated that the hand exoskeleton was generally favorable, observing the absence of significant adverse events after using the equipment. No lesions were reported or observed in the review phase after removal of the equipment. Hence, there were no skin lesions once the therapy was completed. Regarding the presence of pressure areas, all patients showed redness and pressure areas on the skin, which normalized when the exoskeleton was removed. On the other hand, three patients reported pain after the first sessions, however this was associated with the shoulder segment. No pain was reported in the hand, wrist or forearms of the extremity under treatment. No localized fatigue was reported after use the device. Studies available in the literature on the design of devices to recover manual function and restore the quality of life of people with disabilities, present the requirements for the design of these technologies, such as being correctly coupled with the assisted hand, ensure user safety and comfort, be effective in transmitting force and be as affordable and available as possible (Secciani et al., 2011; Sarac et al., 2019). In the study, we used a hand exoskeleton implemented with orthotic adaptations to avoid skin alterations and promote the device safety, which was probably a favorable element in the absence of adverse events in our clinical tests.

### 4.2 Manual function tests

In the present study, most of the tests for manual functionality assessment included in the evaluation protocol could be applied in the study population, with the exception of the 9-HPT and the GPT. These could only be implemented in one patient due to the need of fine motor function, and therefore they were not analyzed. In relation to the analyzed clinical variables, the strength evaluated by dynamometry did not present significant changes for neither grip nor pinch strength. The BBT was used to measure unilateral gross manual dexterity and no statistically significant improvement were found. Significant differences were also not found in the scores coming from the MESUPES, which measures the quality of movement of the hemiparetic arm and hand. Finally, the BI aimed to evaluate the performance in activities of daily living did not suffer significant changes before and after the rehabilitation intervention. In summary, no significant differences were found in any of the assessed manual functional metrics (grip and pinch dynamometry, 9-HPT, GBT, BBT, MESUPES, and BI) when comparing the initial state with the final condition.

One reason of the presented results might be the stroke stage of the four patients included in this study (months since stroke ≥22 months). Although stroke recovery is heterogeneous and it depends on the site and size of the stroke lesion and the person, it has been not yet demonstrated that functional motor improvement will continue beyond 3–6 months after suffering the stroke episode (Gilman, 2006). Chien et al. (2020) presented a meta-analysis in which 11 randomized control trials were reviewed to evaluate the effects of rehabilitation therapy. They concluded that upper-extremity robot-assisted therapy improves motor control, functional independence, muscle tone and performance for those patients who carry out the robot-assisted therapy within the first six months poststroke at post-treatment. However, no significant motor recovery is found beyond that window of time. It has been suggested that it is possible to continue functional motor improvement beyond the 3–6 months using innovative rehabilitation strategies such as neuroaugmentation, electrical or magnetic simulation to enhance cerebral plastic change. However, the efficacy of these technologies has not been verified (Gilman, 2006).

Additionally, the non-significance of the results of the presented study should not have been influenced by the proposed intervention protocol (2 training sessions of 60 min per week for 8 weeks). In the meta-analysis performed by Wu et al. (2021), they concluded that the parameters of the intervention (total training time, number of sessions and training time per session) were not significantly associated with the motor recovery neither in the short term nor in the long term.

Lastly, based on the results, it was identified that the tests selected for the evaluation were focused on the group of patients with the highest degree of voluntary movement, so optimization of the evaluation protocol is required. When (Baker et al., 2011) was designing an assessment protocol for robotic handheld rehabilitation, he developed a three-stage review process as an evidence-based approach to scale selection in stroke rehabilitation study, identifying the scales Stroke Rehabilitation Assessment of Movement, Chedoke Arm and Hand Inventory, and ABILHAND as the best options for his assessment protocol (Baker et al., 2011).

### 4.3 Satisfaction analysis

At the end of the intervention, the QUEST scale was applied to the four study participants. The participants were satisfied with the rehabilitation devices (4.00 ± 0.73). The categories best evaluated by the users were weight (5.00), comfort (5.00), quality of professional services (5.00) and security (4.75). The items dimensions (3.75), adjustments (3.50), durability (3.50), easy to use (3.75), repairs (3.00) and tracing (3.50) presented positive results. However, some users expressed a neutral opinion and therefore, these categories could still be optimized to improve the user experience. Finally, the variables effectiveness of the equipment (3.25) and service delivery (4.00) mostly presented positive results. Nevertheless, there were users who were not very satisfied assessment, which could be related to their high expectations in recovering their manual function completely after the intervention, which was not achieved in the cycle of therapies executed in the present study. Due to the above, the next phases of the study should emphasize modeling the expectations of users, inform that robotic therapies are still in the development phase, for which their effectiveness and optimal procedures and services have not yet been defined. In the same way, the patients who presented low satisfaction for these variables were those who presented the greatest degree of restricted manual function in the admission evaluation and after robotic therapy, which could be associated with the opinion of the users. Therefore, the next phases of the study should emphasize homogenizing the clinical characteristics of the group of participants to improve the quality of the evidence of user satisfaction after robotic therapies.

Due to the limited studies that used validated measure (the QUEST) for the subjective assessment of the rehabilitation and assistive devices, it is difficult to compare the results from different studies (Koumpouros, 2016). After a review of the literature, we have detected four articles reporting the results coming from the QUEST questionnaire on assistive or rehabilitative devices for the hand in stroke patients (see Table 5). Note that these studies only reported eight out of the twelve items of the QUEST; they reported the results of the subscale which the items that assess the assistive device (dimensions, weight, settings, security, durability, easy to use, comfort and effectiveness) and do not include the subscale regarding to the rehabilitation service (service delivery, repairs, quality of service and tracing).

**TABLE 5:** Comparison of the QUEST questionnaire results with other related works.

Device	N patients	Dimensions	Weight	Settings	Security	Durability	Easy to use	Comfort	Effectiveness	Overall
RobHand	4	3.8	5	3.5	4.8	3.5	3.8	5	3.3	4.06 ± 0.72
My-Hero	9	3.2	4	3.4	4.2	3.6	3.6	3.7	3.4	3.63 ± 0.73
Hero	11	2.9	3.4	2.3	4.6	3.4	3.8	2.9	3.4	3.3 ± 0.76
(Dudley et al.,2021)	1	4	4	5	5	5	4	3	3	4.0 ± 0.76
HoMEcare	12	—	—	—	—	—	—	—	—	4.06 ± 0.72

Likert-scale questionnarie: 1- not satisfied at all, 2- not very satisfied, 3- more or less satisfied, 4- quite satisfied, 5- very satisfied.

The QUEST questionnaire was carried out with My-HERO (Yurkewich et al., 2020b) and HERO (Yurkewich et al., 2020a), two robotic gloves that provides five-flexion extension and grip assistance to assist stroke patient to complete activities of the daily living independently. Although both are an assistive device and not a rehabilitation one like RobHand, the three devices assist the flexion and extension motion of the hand fingers independently and they are very similar in terms of the mechanical design. My-Hero reported an overall score of 3.63 ± 0.73, while a HERO 3.3 ± 0.76. Another study performed on a rehabilitation hand exoskeleton reported the QUEST scores coming from only one stroke patient with an overall score of 4 (Dudley et al., 2021). HoMEcare aRm rehabilitation (MERLIN), a robotic device for upper-limb rehabilitation (shoulder, elbow, wrist and fingers), was also evaluated using the QUEST questionnaire (Rozevink et al., 2021). They only reported the overall score of 3.9 ± 0.39. It must be taken into account that MERLIN is an upper-limb robot with a total of 7 degrees of freedom and therefore, direct comparisons with the Robhand exoskeleton cannot be made. The four stroke patients included in the present study reported an overall score of 4.06 ± 0.72, indicating that the users are satisfied with the technology. It can be therefore concluded that the user satisfaction results using the RobHand exoskeleton are above average.

## 5 Conclusion

This study included four chronic stroke patients and evaluated the security, rehabilitation capabilities and usability of the RobHand: a hand robotic device which assist the flexion and extension of the fingers independently for neuromotor rehabilitation. The robotic system was tested and it is safe. It should be stand out the absence of significant adverse events and risks for its implementation in stroke patients with motor disabilities. However, no significant improvements were found in manual motor function. Therefore, it can not be concluded the rehabilitation effectiveness of RobHand in chronic stroke patients. Lastly, it is highly important to remark that the users were satisfied with the RobHand exoskeleton, and therefore, it is highly probable that this technology will be accepted in the field.

The present study had limited number of stroke patients, recruited from a single geographic location and no control group was established. First, it would be advised that future studies include a control group and a larger sample size. Additionally, they should consider the high influence of the stroke stage on the motor recovery and hence, change the target sample population from chronic to subacute stroke patients. However, taking into account that the Corporación de Rehabilitación Club de Leones Cruz del Sur mainly treats chronic patients, another approach will be to evaluate the effects of using the RobHand exoskeleton combined with innovative technologies such as neuromuscular electrostimulation, given that it has reported a possible increasing of functional and motor skills, together with a higher prevalence of gaining this improvement over time. Lastly, if new trials are performed with chronic stroke patients, the evaluation protocol should be optimized for this target population.

Likewise, it would be advisable to incorporate the RobHand as a rehabilitation tool in patients with other diseases of the nervous and musculoskeletal system such as spinal cord injury, peripheral neural injuries, or in the rehabilitation of traumatic and post operative musculoskeletal injuries of the hand, in search of improvements in the manual function recovery.

## Data Availability

The raw data supporting the conclusion of this article will be made available by the authors, without undue reservation.

## Abbreviations

CVA, Cerebrovascular accidents; ROM, Range of motion; MCP, Metacarpophalangeal; PIP, Proximal interphalangeal; 9-HPT, Nine Hole Peg Test; GPT, Grooved pegboard test; BBT, Box and blocks test; MESUPES, Motor evaluation scale for upper extremity in stroke patients; BI, Barthel Index; QUEST, Quebec user evaluation of satisfaction with assistive technology.
