# Supplementary material for: Hand rehabilitation based on the RobHand exoskeleton in stroke patients: A case series study
Source: Front Robot AI. 2023 Mar 22;10:1146018. doi: 10.3389/frobt.2023.1146018 (PMC10073561; doi:10.3389/frobt.2023.1146018)
Supplement: Supplementary file 1 [file DataSheet1.PDF]

frontiers

Supplementary Material

1 INTRODUCTION

Clinical studies have found significant improvement in hand motor function when performing robotic rehabilitation (Carmeli et al., 2011; Ueki et al., 2012; Kutner et al., 2010). The RobHand (Robot for Hand Rehabilitation) is a hand robotic rehabilitation platform, which provides assistance for flexion and extension movements of the hand fingers. As any new developed rehabilitation technology, it is necessary to evaluate its effectiveness in clinical settings. However, prior performing a clinical trial, we tested it with healthy subjects to evaluate safety, user satisfaction and the intervention protocol itself. Therefore, the present report presents the results of a pilot study which aims to gather information about the experience of subject without pathology after training with the RobHand exoskeleton, particularly information about safety aspects and user satisfaction.

2 METHODS

2.1 Settings

The present pilot study was set at the Rehabilitation Center Club de Leones Cruz del Sur in Punta Arenas (Chile). It was approved by the Research Ethics Committee of the Club de Leones Cruz del Sur Rehabilitation Corporation.

2.2 Participants

An interventional, non-controlled study was carried out with a sample selected for convenience. This study included 4 participants healthy participants (3 men and 1 women), all legal age and workers at the Rehabilitation Center Club de Leones Cruz del Sur. Demographic data of the participants is shown in Table S1. To ensure privacy, each participant was assigned an alphanumeric code.

Table S1. Participants demographics

ID Participant	Gender	Age	Dominance
LR6MAG	M	35	Right
RUGDCT	F	29	Right
RV6PA6	M	39	Right
WY9ZQZ	M	34	Right

The inclusion and exclusion criteria for the study is summarized in Table S2. All participants signed the informed consent form.

2.3 Rehabilitation system

The RobHand (Robot for Hand Rehabilitation) is an exoskeleton-type electromechanical device (Figure S1), which is attached to the patient's hand and provides assistance for performing different types of finger

Table S2. Inclusion and exclusion criteria

Inclusion criteria	Exclusion criteria
Volunteers without musculoskeletal disorders of the upper extremity	Musculoskeletal disorders of the upper extremity
Volunteers without neurological pathology	Presence of central nervous system diseases
Age between 18 and 65 years	Hand or upper extremity pain
Ability to follow instructions	Does not sign the informed consent form

movement rehabilitation therapies. The RobHand platform was developed by the University of Valladolid, Spain (Cisnal et al., 2018).

The exoskeleton is composed of five independent subassemblies that are placed on a platform which is located on the back of the hand, with the exception of the thumb subassembly that is mounted on a separated module connected to the hand support platform through a linkage device. Each subassembly includes a linkage underactuated mechanism that transmits the movement of the linear actuator L12-30-100-6-I (Actuonix Motion Devices Inc., Victoria, BC, Canada) to a double-ring. The exoskeleton is attached to the proximal and distal phalanges through the double-rings. Hence, the proposed linkage mechanism allows to control flexion and extension angles of the metacarpophalangeal (MCP) and proximal interphalangeal (PIP) joints of each finger with a single linear actuator. Therefore, the motion of the MCP and PIP joints of each finger is kinematically coupled. The use of this linkage mechanism reduces size, weight and cost of the exoskeleton due to the reduction in the number of actuators used. Furthermore, the mechanical structure has been optimized to cover the ROM of a healthy human hand with a 30 mm stroke actuator. More precisely, the index finger can reach a maximum hyperextension movement of 8º and -5º at the MCP and PIP angles and a maximum flexion of -63º and -76º at the MCP and PIP joints, respectively.

The double-rings ease the donning and doffing procedure of the exoskeleton, which is critical for patients suffering from hand spasticity. The custom double-rings made of flexible material (Filaflex 82a) material are placed on each finger, and then they are jointed to the corresponding linkage mechanism. Furthermore, due to the special mobility characteristics of the thumb, the exoskeleton integrates a simple mechanism that provides an easy adaptation of the thumb subassembly to the exoskeleton and therefore, allowing to achieve comfortable movements of the thumb, regardless its size and length. The hand exoskeleton is adjusted on the hand of the patient using two Velcro straps joint to the hand support platform and placed around the hand palm and wrist. The forearm of the participants is supported on a semisoft wedge-shaped platform. The hand exoskeleton structure was manufacturing in 3D printing using of PLA and it weights approximately 450 gr (Moreno-SanJuan et al., 2021).

The five linear actuators are independently controlled by a TMS300F28069M microcotnroller (Texas Instruments, Texas, EEUU) using a 0-5V interface and are powered by 6 V DC. A custom-made motor driver PCB allows to proper control the actuators with the microcontroller and provides a 20-pin single connector to interface the hand exoskeleton to the electronic box. All the electronics are housed in a 3D-printed electronic box. The electronic box has a reset button, a 6 V DC jack power connector, an on/off switch, a visual indicator light and a kill-switch for security reasons (Cisnal et al., 2021).

The RobHand platform allows patients to perform passive training exercises, which involves the continuous repetition of finger flexion and extension movements at three predefined velocities. The hand exoskeleton is controlled by a windows-based application. Before starting a rehabilitation session, the therapist must log in to the application using their credentials and select the patient among those registered in the application or create a new one if it does not exist. Then, the therapist must select on the user interface which type of exercise to perform and configure it by specifying the number of repetitions and

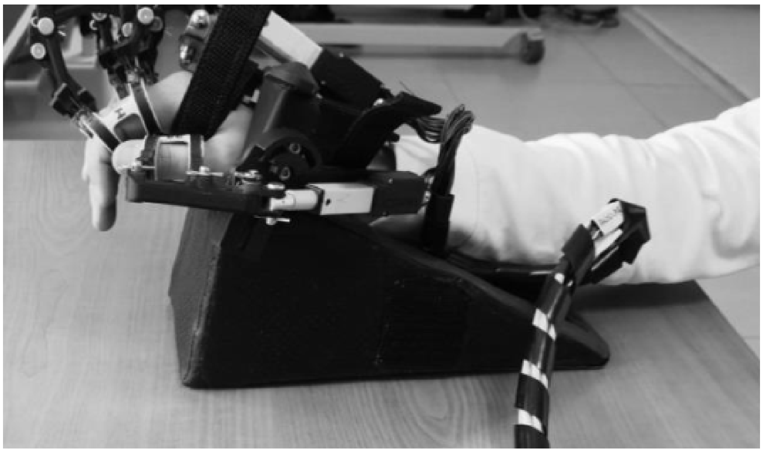

Figure S1. Robhand Exoskeleton

velocity (low, medium and high). Four types of rehabilitation exercises are available: (i) Squeeze oranges - flexion and extension of the hand fingers with the aim making orange juice (ii) Hand opening and closing - flexion and extension of the five hand fingers simultaneously (iii) Fingers opening and closing - flexion and extension of hand fingers individually (iv) Pinch grip and precision grip - flexion and extension of the thumb against the four fingers or against the index finger. The therapist can also define a comfortable ROM (maximum and minimum MCP angles for each finger) for performing therapies according to the patient's residual skill (Cisnal et al., 2022). All data is store in a local database and the therapist can review the therapy history of each patient.

2.4 Intervention

The intervention consisted of 2 training per week for a total of 16 sessions using the RobHand exoskeleton on the right hand. These sessions occurred between October 2020 and March 2021. Each training session was divided into 6 consecutive stages: equipment installation, hand opening and closing, squeeze oranges, precision grip, pinch grip and equipment removal.

Stage 0 - Equipment installation:

The participant is asked to sit in an ergonomic chair with his arm flexed at 90º, with his forearm resting on a semisoft wedge on a table, to leave the hand free for the movements performed by the exoskeleton. The installation, configuration and positioning of the exoskeleton was performed by an occupational therapist with experience in robotic rehabilitation.

Stage 1 - Hand opening and closing:

The training began when the trader gave the start indication, at this point the RobHand performed the opening-closing actions for the participant. In this stage, after each cycle, a 2-second pause was made, contemplating a total of 75 repetitions per session. The duration of this stage is 5 minutes and 7 seconds.

Stage 2 - Squeeze oranges:

Prior to performing this exercise, audiovisual material is shown indicating how to perform the exercise correctly. This material is available in the exoskeleton software. The exercise is performed in sync with the audiovisual material, where you see visually how a hand is squeezing oranges meanwhile the RobHand is performing this action with the hand of the participant (Figure S2**A**). After each repetition the action

is pausing for 4 seconds. This stage had a total duration of 4 minutes and 11 seconds, and there were performed 19 repetitions of the exercise.

Stage 3 - Precision grip:

Regarding this task, the preconfiguration provided by the RobHand software was used to perform fine pincer movements, grasping objects with the thumb and index finger. We worked 2x2 cm2 cubes .The instructions given by the evaluator were as follows: “I am going to ask you to use this boxes (the instructor hold the box with 200 cubes near dominant hand of the participants) to monitor the movements of pincer, so you need to grab any cube from the box and leave it on the other box ” (Figure S2**B**). This task had a total duration of 5 minutes and 11 seconds, the type of grip for the pencil used was big, between each closing and opening action of fine grasps, pauses of two seconds were made, the speed of the exercise was high and completed a total of 75 repetitions.

Stage 4 - Pinch grip:

In the present task, cylinders of two different sizes with a variation of 1 cm in diameter were used. A table with pins was placed so that the participant could position the cylinders according to size. The evaluator left the cylinders next to the participant so they can easily access to them. Afterwards he was given the following instruction: “I am going to ask you to take each one of the cylinders for each hand opening movement and stack them in the spaces of the board. It is important that you start with the smallest ones and continue with the bigger ones” (Figure S2**C**).

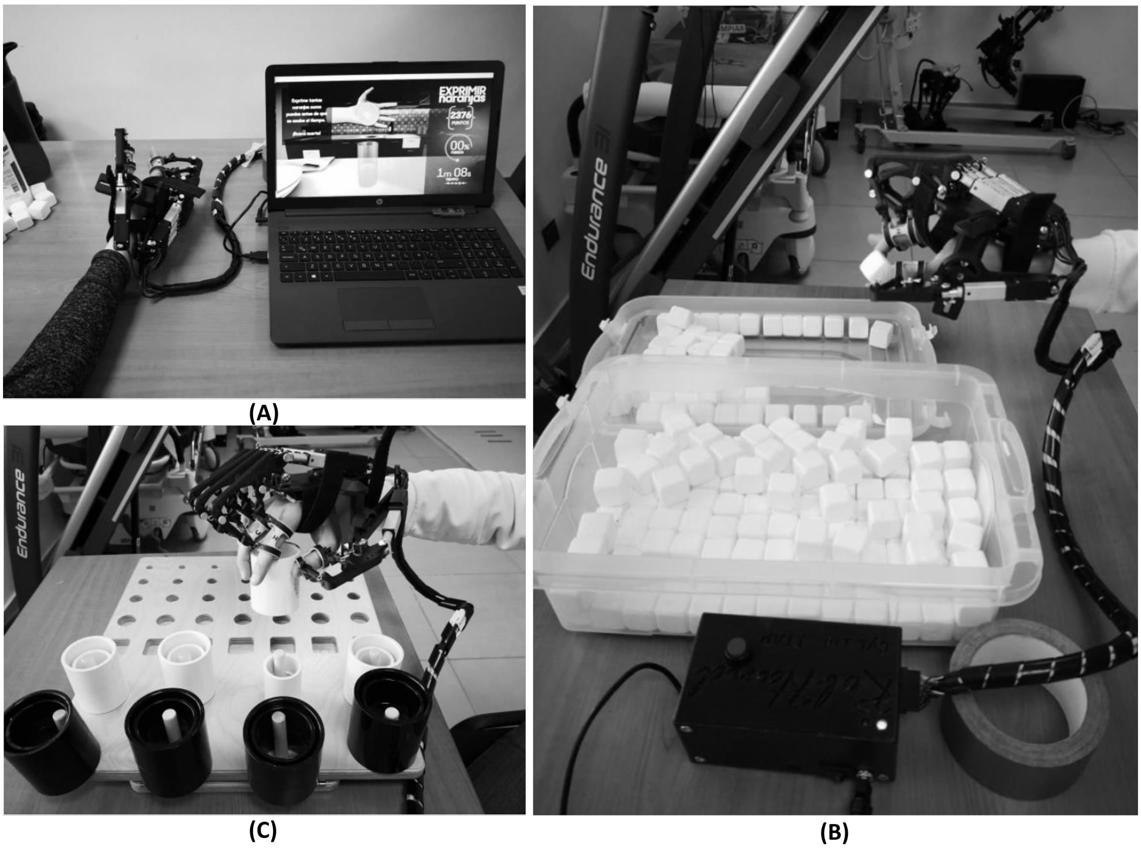

Figure S2. Participant performing the intervention. **(A)** Stage 2 - Squeeze oranges **(B)** Stage 3 - Precision grip **(C)** Stage 4 - Pinch grip

This task had a total duration of 5 minutes and 11 seconds, with a total of 20 repetitions. The grip for the cones was great and the speed of the task was medium, with a duration between each coarse pincer movement of 4 seconds.

Stage 5 - Equipment removal:

The removal of the exoskeleton was performed by an occupational therapist with experience in robotic rehabilitation. The evaluator after withdrawal the equipment, checked adverse events like pressure points, skin problems or pain and registered them. This stage had a time close to 5 minutes.

2.5 Assessments

The evaluation of the device safety, manual function and user satisfaction was carried out. Different instruments were used for manual function assessment: dynamometry for grip and pinch strength assessment, Nine Hole Peg Test (9-HPT), Grooved Pegboard Test (GPT). Manual function assessments were performed before (participant's baseline or T0) and after the intervention with the RobHand robotic exoskeleton (post-intervention or T1). Safety tests were carried out after each training session. Furthermore, all participants fulfilled the Quebec User Evaluation of Satisfaction with Assistive Technology (QUEST 2.0) questionnaire after the intervention (T1).

Safety test

The clinician asked the participant after every training session whether he/she has suffered pain or localized fatigue. The clinician also visually checks whether there are presence of cutaneous lesions or pressure zones in the skin.

Grip strength assessment

A Jamar hydraulic hand dynamometer (JAMAR, hydraulic, model 12-0600, 5lbs or 2 kg gradations, Pennsylvania, USA) was used to measure grip strength. It allows to evaluate forces up to 200 lbs (90Kg). This test allows to evaluate the functional integrity of the upper extremity through the force exerted when squeezing the hand and therefore, to identify the loss of physiological muscle function. The participant is asked to grasp the resistance of the handle, place his shoulder in abduction and with neutral rotation. Additionally, the elbow must be flexed at 90º and with the forearm in a neutral position, the wrist between 0º and 30º dorsiflexion, and between 0º and 15º ulnar deviation. Grip measurements were repeated three times and the average value is reported (Ong et al., 2017; Armando et al., 2012).

Pinch strength assessment

The force exerted with the index finger and thumb is assessed using a Jamar hydraulic pinch gauge (JAMAR, hydraulic pinch, model 12-0601, gradations 1lbs or 0.5kg gradations, Pennsylvania, USA) (Figure S3**A**). It allows to evaluate forces up to 50 lbs (30 Kg). The measurement is standardized in its procedure according to publications of literature. The thumb was positioned on top of the pinch gauge's force pad and the index fingertip was positioned underneath. The researcher supported the gauge and asked the participant to grip and pinch with their maximum strength. The measurement was performed three times (Gilbertson and Barber-Lomax, 1994).

Nine Hole Peg Test (9-HPT)

9-HPT seeks to evaluate the dexterity of the fingers, for which a board and nine pegs are used (Figure S3**B**). The participant must place the 9 pegs on the board and then remove all of them, using the dominant hand (Oxford Grice et al., 2003).

Grooved Pegboard Test (GPT)

GPT allows to evaluate a variety of psychomotor skills, among which is present: fine motor skills, motor speed and hand-eye coordination. The test consists of placing 25 pegs, with a key along one side, on a board with a 5 by 5 matrix, that is, 25 holes, which have different orientations, so the pegs must be rotated to match the hole. The test is performed with the affected upper limb. This test is timed. If the tested person is not able to match all pegs in 5 minutes, the tester counts the number of matched pegs on the board (Tolle et al., 2020).

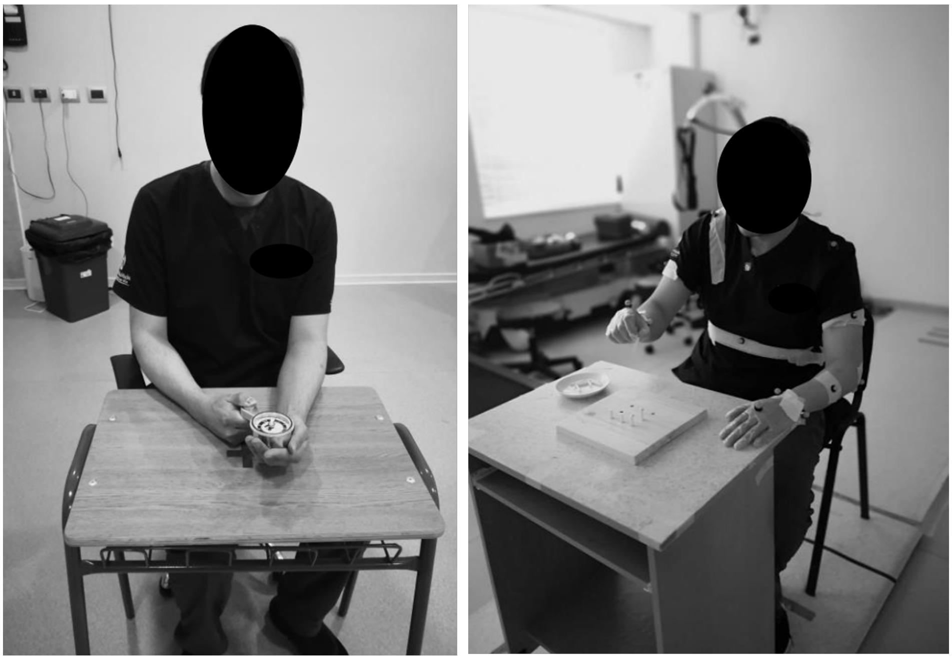

(A) (B)

Figure S3. Manual function assessments. **(A)** Pinch strength assessment **(B)** Nine Hole Peg Test.

Evaluation of user satisfaction with assistive technologies in Quebec (QUEST 2.0)

It is a self-administered questionnaire that allows to consider personal aspects related to the use of a device, in order to assess user satisfaction when using an assistive device. This can be applied to adolescents, adults and older adults. This questionnaire consists of 2 sub-scales; the first evaluates 8 aspects related to the assistance team, while the second evaluates 4 aspects related to the services provided while the participant uses the device. The responses are rated from 1 to 5, where 1 means not at all satisfied and 5 means very satisfied (Demers et al., 2000).

2.6 Statistical Analysis

Statistical analyses were performed using the R Statistical Software with the alpha level set to 0.05 for statistical significance. A mixed model analyses of variance (ANOVA) were performed to evaluate changes in baseline (T0) and post-intervention (T1) on the scores of the performed manual function tests. The models include one fixed explanatory variable (T0 or T1 intervention stage) and a random effect variable (participant). The unadjusted p-values are reported for each test.

3 RESULTS

The results of this intervention have been divided into 3 edges; safety tests, manual function tests and satisfaction analysis.

Safety Test

The safety results of the four participants after using the RobHand exoskeleton (Table S3 showed that there was an absence of adverse events during the study period. However, two of the participants reported feeling pressure on the skin once the equipment was removed, and one user reported one user reported localized fatigue.

Table S3. Results of the safety test after using the RobHand: Presence of pain, cutaneous lesions, skin pressure zones and localized fatigue

ID Particpant	Pain	Cutaneous lesions	Skin pressure zones	Localized fatigue
LR6MAG	No	No	Yes	No
RUGDCT	No	No	Yes	Yes
RV6PA6	No	No	No	No
WY9ZQZ	No	No	No	No

Manual Function Tests

The measured grip and pinch force and the results of the 9-HPT and GPT for each participant before (T0) and after the intervention (T1) are shown in Table S4. The mean generated grip force for the four participants before the intervention was 50.3±16.93 kg and after the intervention 46.78±16.95 kg. No statistically significant differences are observed when comparing T0 and T1 (Chi square = 0.33333, p = 0.5637). Respect to the pinch force, the mean of the four participants before the intervention was 10.58±2.04 kg and after the intervention 10.06±2.04 kg. No statistically significant differences are observed when comparing T0 and T1 (Chi square = 0.083333, p = 0.7728).

Table S4. Manual function results obtained before and after the intervention

ID Particpant	Grip(kg)		Pinch(kg)		9-HPT (s)		GPT (s)	
	T0	T1	T0	T1	T0	T1	T0	T1
LR6MAG	59.6	24.1	11.0	7.4	12.0	10.9	46.3	53.1
RUGDCT	25.3	60.3	7.8	12.0	11.4	13.9	54.8	49.7
RV6PA6	61.7	43.5	12.7	9.7	12.3	16.4	54.6	52.3
WY9ZQZ	54.6	59.2	10.8	11.1	15.6	14.8	50.4	56.1
Mean	50.30	46.78	10.58	10.06	12.82	14.00	51.53	52.80
SD	16.93	16.95	2.04	2.00	1.89	2.31	4.03	2.64

The average of the results for the 9-HPT before the intervention was 1.98±1.89 s and after the intervention 14.00±2.31 s. No statistically significant differences are observed when comparing T0 and T1 (Chi square = 0.33333, p = 0.5637). Regarding the results of the GPT, the average before the intervention was 51.53±4.03 s and after the intervention 52.8±2.64 s. No statistically significant differences are observed when comparing T0 and T1 (Chi square = 0.083333, p = 0.7728). No statistically significant differences are found in neither of the four performed manual tests, which means that there is no relevant change between the evaluation before and after training.

User satisfaction

After completing 16 training sessions, the QUEST questionnaire was performed by all participants (Figure S4). In relation to the dimensions of the team, 25% consider themselves more or less satisfied with them, 50% quite satisfied and 25% very satisfied. 75% were very satisfied and 25% more or less satisfied with the weight of the robot. Regarding the adjustments, that is, if the team adapts to its context, 75% were neutral and 25% very satisfied. In the case of the security variable, all of them feel satisfied with the use of the hand exoskeleton. In relation to ease of use, that is, if the subjects consider it easy to use the equipment, 75% were satisfied and 25% strongly satisfied. Regarding the effectiveness of the team, 25% were quite satisfied and 75% strongly satisfied. This means that all of the users consider a normal or high effectiveness in the use of the equipment in relation to the objective set in the exercise. The levels of satisfaction regarding the delivery of service during the use of the equipment, that means that the information provided was clear and concise, show a result of 25% of highly satisfied users, 50% quite satisfied and 25% more or less satisfied. With respect to user confidence in the use of RobHand in clinical rehabilitation applications, 25% were neutral, 50% were quite satisfied and 25% were very satisfied. Furthermore, 75% were quite satisfied and 25% strongly satisfied with the robot speed. In relation to learning, 75% were quite satisfied and 25% were strongly satisfied in terms of how easy is to learn to use the robotic platform. Finally, regarding equipment aesthetics, 25% were neutral, 50% were quite satisfied and 25% were strongly satisfied.

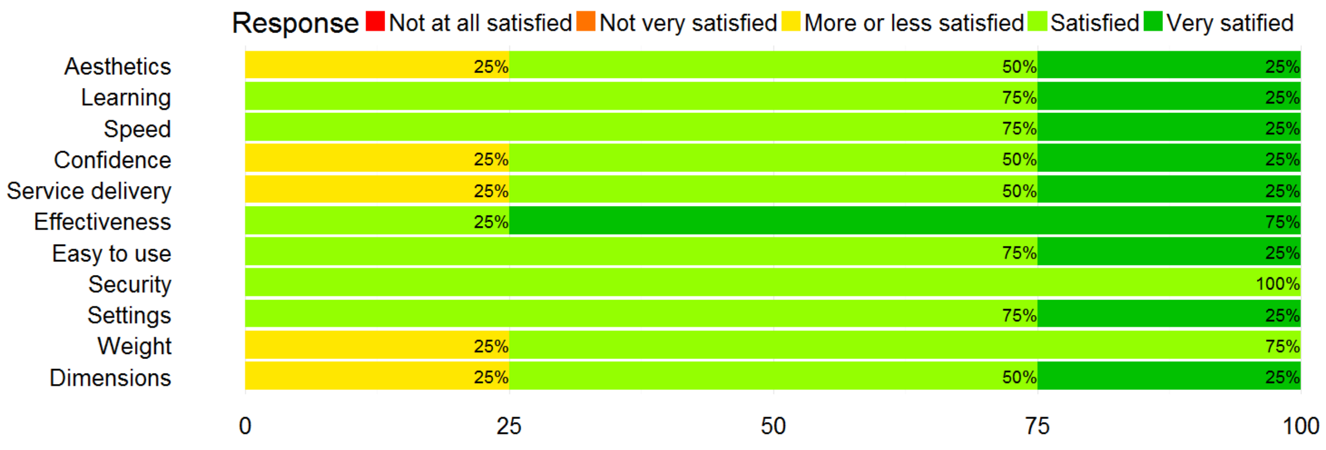

Figure S4. User satisfaction results from the QUEST questionnaire.

4 DISCUSSION

The objective of this pilot study was to know the experience of subjects without pathology after training with the RobHand device, identifying the satisfaction of the participants with respect to the use of the exoskeleton and safety aspects. The satisfaction of the users with the use of the hand exoskeleton was favorable, with an absence of pain after using the device. At the same time, no lesions were reported or observed in the revision phase after the removal of the equipment; therefore, there were no skin lesions once the therapies were finished. Regarding the presence of pressure zones, two participants reported having experienced them. This was due to the fact that a suitable size of the flexible double-ring was not found for these participants. Likewise, one participant reported localized fatigue after the use of the device due to the absence of stabilization in the wrist flexion-extension plane.

Taking into consideration these results and in order to improve the safety of the device for a future clinical trial, changes on the design of the exoskeleton are proposed for future studies. First, the double-rings made of flexible material (Filaflex 82a) will be replaced with custom double-rings made of ORFICAST® thermoplastic material in order to better adjust the size of the ring to each participant and avoid pressure areas. Secondly, the semisoft wedge-shaped platform which is used for forearm support with be replaced for a flexible splint which secures the position of the wrist.

It is important to emphasize that the present report was based on a pilot study. Therefore, no changes in manual function could be observed. However, it is noteworthy the absence of adverse events during the training sessions, where each participant managed to finish them with the exoskeleton in an optimal manner and without reporting complaints.

5 CONCLUSION

In the present report, there was no evidence of risks that outweighed the benefits of the use of the RobHand robotic platform, highlighting the presence of more benefits than risks for its implementation in subjects. In turn, it demonstrated that the use of the RobHand exoskeleton in the hand of subjects without pathology is safe for repetitive use. Considering the outcomes of the safety evaluation, two minor changes in the design have been proposed to improve safety and ergonomics for future trials. It is important to note that the study had a limited number of participants, recruited from a single geographic location and no control group was established. Hence, future studies should evaluate the effects of the use of the RobHand exoskeleton as a rehabilitation tool in patients with central nervous system diseases such as stroke, looking for improvements in the recovery of manual function.

REFERENCES

Armando, R. C. J., del Carmen, L., Valentín snachez, G., Datta Banik, S., and Argáez S., J. (2012). Dinamometria de manos en estudiantes de merida, méxico [hand dynamometry in students from merida, mexico]. *Revista chilena de nutricion* 39, 45–51. doi:10.4067/S0717-75182012000300007

Carmeli, E., Peleg, S., Bartur, G., Elbo, E., and Vatine, J.-J. (2011). Handtutor^{TM} enhanced hand rehabilitation after stroke - a pilot study. *Physiotherapy Research International* 16, 191–200. doi:10.1002/pri.485

Cisnal, A., Lobo, V., Moreno, V., Fraile, J.-C., Alonso, R., and Pérez-Turiel, J. (2018). Robhand, un exoesqueleto de mano para la rehabilitacion neuromotora aplicando terapias activas y pasivas [robhand, a hand exoskeleton for neuromotor rehabilitation using passive and active therapies]. In *Actas de las XXXIX Jornadas de Automatica, Badajoz, 5-7 de Septiembre de 2018*. 34, 41. doi:10.17979/spudc.9788497497565.0034

Cisnal, A., Moreno-SanJuan, V., Fraile, J., Turiel, J., de-la Fuente, E., and Sanchez-Brizuela, G. (2022). Assessment of the patient's emotional response with the robhand rehabilitation platform: A case series study. *J. Clin. Med.* 11, 4442. doi:10.3390/jcm11154442

Cisnal, A., Pérez-Turiel, J., Fraile, J.-C., Sierra, D., and de la Fuente, E. (2021). Robhand: A hand exoskeleton with real-time emg-driven embedded control. quantifying hand gesture recognition delays for bilateral rehabilitation. *IEEE Access* 9, 137809–137823. doi:10.1109/ACCESS.2021.3118281

Demers, L., R., W.-L., and B., S. (2000). Item analysis of the quebec user evaluation of satisfaction with assistive technology (quest). *Assist Technol.* 12, 96–105. doi:10.1080/10400435.2000.10132015

Gilbertson, L. and Barber-Lomax, S. (1994). Power and pinch grip strength recorded using the hand-held jamar® dynamometer and b+l hydraulic pinch gauge: British normative data for adults. *British Journal of Occupational Therapy* 57, 483–488. doi:10.1177/030802269405701209

Kutner, N. G., Zhang, R., Butler, A. J., Wolf, S. L., and Alberts, J. L. (2010). Quality-of-life change associated with robotic-assisted therapy to improve hand motor function in patients with subacute stroke: a randomized clinical trial. *Physical therapy* 90, 493–504. doi:10.2522/ptj.20090160

Moreno-SanJuan, V., Cisnal, A., Fraile, J.-C., Pérez-Turiel, J., and de-la Fuente, E. (2021). Design and characterization of a lightweight underactuated raca hand exoskeleton for neurorehabilitation. *Robotics and Autonomous Systems* 143, 103828. doi:10.1016/j.robot.2021.103828

Ong, H. L., Abdin, E., Chua, B. Y., Zhang, Y., Seow, E., Vaingankar, J. A., et al. (2017). Hand-grip strength among older adults in singapore: a comparison with international norms and associative factors. *BMC geriatrics* 17, 1–11. doi:10.1186/s12877-017-0565-6

Oxford Grice, K., Vogel, K., Le, V., Mitchell, A., Muniz, S., and Vollmer, M. (2003). Adult norms for a commercially available nine hole peg test for finger dexterity. *Am J Occup Ther.* 57, 570–3. doi:10.5014/ajot.57.5.570

Tolle, K. A., Rahman-Filipiak, A. M., Hale, A. C., Andren, K. A. K., and Spencer, R. J. (2020). Grooved pegboard test as a measure of executive functioning. *Applied Neuropsychology: Adult* 27, 414–420. doi:10.1080/23279095.2018.1559165

Ueki, S., Kawasaki, H., Ito, S., Nishimoto, Y., Abe, M., Aoki, T., et al. (2012). Development of a hand-assist robot with multi-degrees-of-freedom for rehabilitation therapy. *IEEE/ASME Transactions on Mechatronics* 17, 136–146. doi:10.1109/TMECH.2010.2090353
